# Reasons for not seeking alcohol treatment among a sample of Florida adults with HIV who perceived the need for treatment

**DOI:** 10.1186/s13722-024-00491-5

**Published:** 2024-10-04

**Authors:** Christina E. Parisi, Nanyangwe D. Siuluta, Shantrel S. Canidate, Robert L. Cook, Yan Wang, Maya Widmeyer, Charurut Somboonwit, Jessy G. Dévieux, Natalie Chichetto

**Affiliations:** 1 Department of Epidemiology, College of Public Health and Health Professions, College of Medicine, University of Florida, Gainesville, FL USA; 2 Unconditional Love Incorporated, Melbourne, FL USA; 3 Department of Internal Medicine, Morsani College of Medicine, University of South Florida, Tampa, FL USA; 4 Department of Health Promotion & Disease Prevention, Robert Stempel College of Public Health & Social Work, Florida International University, Miami, FL USA

## Abstract

**Background:**

A minority of people who need alcohol treatment receive it. Unhealthy alcohol use is common among people with HIV (PWH) and can lead to negative health outcomes. The aims of this multi-methods study are to (1) quantitatively describe the prevalence, psychosocial characteristics, and demographic traits of a sample of PWH currently receiving HIV care in Florida who had a self-reported need for alcohol treatment but did not seek care and (2) qualitatively explore reasons why PWH did not seek treatment.

**Methods:**

PWH enrolled in the Florida Cohort Study between October 2020 and February 2023 who had drinking history (*N* = 487) completed a cross-sectional survey that asked if there was a time when they recognized they needed help for their drinking but did not seek it. If yes, they were asked an open-ended follow-up question about reasons why they did not seek care. Demographic and behavioral differences between those who did and did not endorse a time when they needed alcohol treatment were determined using multivariable logistic regression, while qualitative data were analyzed with thematic analysis based in the Social-Ecological Model to assess reasons for not seeking care at the individual, social, and systems levels.

**Results:**

A quarter of PWH (*n* = 129) with lifetime drinking indicated a time they needed care but did not seek it. Patients who endorsed a time where they perceived the need for treatment but did not seek it were more likely to endorse current at-risk drinking and a history of ever trying to reduce their drinking or formally seek professional alcohol treatment. The most common reasons participants did not seek care were individual level factors and included shame, denial, fear, wanting to do it on their own, not feeling ready, and not wanting to seek care.

**Conclusions:**

PWH experienced barriers largely at the individual level that prevented them from seeking alcohol treatment despite a recognized need, though many eventually sought care. Providers and public health professionals should consider helping to address various barriers, particularly internal barriers, when designing interventions to help PWH seek care.

## Introduction

Data suggest a high prevalence of unhealthy alcohol use, defined as levels of drinking above recommended levels and including Alcohol Use Disorder (AUD) [1], among people with HIV (PWH). Some estimates of unhealthy alcohol use among PWH are as high as 42% [2]. Moreover, unhealthy alcohol use is associated with higher risk of HIV transmission, lower adherence to HIV antiretroviral therapy (ART), lower viral suppression, and increased risk for a wide range of comorbid conditions and all-cause mortality [3–11].

A minority of both PWH and people without HIV (PWOH) who need alcohol treatment to address their unhealthy drinking and/or AUD receive it. One study estimated the annual probability of receiving alcohol treatment among PWH with unhealthy alcohol use at 45% [12]. For many, limited treatment options and a variety of barriers prevent them from seeking care even if they recognize they need it. Such barriers include financial difficulties, shame and stigma of alcohol use, attitudes and beliefs, or not knowing how or where to seek treatment [13–16]. Those with HIV might have unique reasons for not seeking alcohol treatment, such as using alcohol to cope with their HIV diagnosis [17] or treating co-occurring conditions such as pain [18]. Moreover, there are established racial and ethnic disparities in substance use treatment seeking behavior, with those identifying as non-Hispanic Black or Hispanic/Latino having lower rates of treatment utilization in multiple studies and Hispanic/Latino individuals having a lower rate of perceived need for treatment as compared to non-Hispanic White individuals [19–21]. It is important to understand barriers to alcohol treatment seeking behaviors among PWH so interventions can be designed to overcome them and connect PWH to the treatment they need.

The Social-Ecological Model (SEM) states that health is affected by characteristics that operate at the individual, social, and systems level as well as by the interaction of characteristics at these different levels [22–24]. It has been applied to the study of a variety of health conditions and behaviors including understanding barriers to HIV care [25, 26]. Applying the SEM model to a study examining reasons why PWH did not seek alcohol use treatment despite thinking they had the need for it can elucidate gaps at the individual, social, and systems-level, allowing for more targeted resource distribution and intervention designs. We hypothesize that reasons for not seeking treatment despite a perceived need will exist at levels inside and outside the individual, which is why we have chosen to base this work in the SEM.

Understanding reasons why people might not seek alcohol use treatment, even when they recognize a need for it, can help leverage existing resources, fill in gaps in care for PWH experiencing alcohol-related issues, help providers give appropriate treatment to their patients, and allow public health professionals to create interventions that meet patients’ needs. This study seeks to quantitatively understand who these underserved PWH are and, through a qualitative analysis of open-ended survey responses, why PWH who self-reported a need for alcohol treatment did not seek care. The study aims are to (1) quantitatively describe the prevalence, psychosocial characteristics, and demographic traits of a sample of PWH currently receiving HIV care in Florida who had a self-reported need for alcohol treatment but did not seek care and (2) qualitatively explore and identify reasons why people did not seek treatment, using the SEM as a framework for our analysis.

## Methods

### Study and study participants

The study sample came from the Florida Cohort study. The goal of the Florida Cohort, a combined longitudinal and cross-sectional survey study, is to investigate healthcare utilization and outcomes among PWH throughout the state to improve healthcare services and overall health [27]. Participants were recruited from private and public healthcare settings in six counties (Alachua, Brevard, Hillsborough, Marion, Miami-Dade, and Palm Beach); three of these (Hillsborough, Miami-Dade, and Palm Beach) are priority areas for the Ending the HIV Epidemic Initiative [28, 29] and all are considered urban as of the 2020 Census [30]. None of the recruitment settings have integrated alcohol and HIV treatment, so participants would need to get referrals for alcohol treatment from their providers or seek treatment on their own. Most participants were able to complete the surveys individually on their mobile device, personal computer, or on paper, although some were administered by a trained research assistant at the participant’s request. Eligibility criteria were: 18 or older, living with HIV, and currently receiving HIV care in Florida. Surveys were available in English, Spanish, and Haitian Creole. Pregnant individuals were not excluded, and pregnancy was not assessed during enrollment or screening for the survey so the number of pregnant participants is unknown. Participants were eligible for the present analyses if they completed the baseline questionnaire between October 2020 and February 2023 (*n* = 544). Of these, 487 (90%) had any drinking history and answered the survey questions of interest (see *Measures* below), making up the final sample.

### Measures

Baseline self-reported demographics included, age, race/ethnicity, gender identity, education, and employment. Self-report of past 12-month substance use included cocaine/crack cocaine, heroin, stimulants including amphetamines, prescription opioids not used under medical supervision, ecstasy/MDMA, hallucinogens, poppers (amyl nitrate), or other drugs; cannabis was not included in this analysis. Alcohol use was assessed using the Alcohol Use Disorders Identification Test (AUDIT-C), which is used to identify people who have unhealthy drinking patterns and potential AUD [31–33]. Participants assigned female at birth with an AUDIT-C score of 3 or more or those who were assigned male at birth with an AUDIT-C score of 4 or more were categorized as being at-risk for unhealthy drinking, while those with alcohol use below these thresholds were categorized as being not at-risk for unhealthy drinking [33]. Participants with no alcohol use in the preceding 12 months were categorized as abstinent or “no alcohol use”. Anxiety symptoms were measured with the Generalized Anxiety Disorder-7 (GAD-7) [34] and depression symptoms were measured with the eight-item Patient Health Questionnaire (PHQ-8) [35]; a score of 10 or more on these measures was classified as having present symptoms. To assess history of ever attempting to reduce drinking, participants were asked: “Have you ever tried to quit or cut back on your drinking?” with the potential answer choices of “Yes” or “No.” To assess history of seeking professional alcohol treatment, participants were given a list of alcohol treatment strategies (Alcoholics Anonymous [AA], counseling or therapy, inpatient or outpatient treatment, medication, or self-monitoring with a device such as a breathalyzer or wrist monitor) and asked if they had ever used that strategy.

After a prompt, “There are many different reasons why people who drink may not seek help. We are interested in learning about barriers to alcohol treatment that you have either faced or worried about facing,” participants were asked: “Was there ever a time when you thought you should seek help for your drinking, but you didn’t go?” The answer options were “Yes” or “No.” If the participant answered “Yes,” they were asked two more questions: “Did this happen during the last 12 months?” to which participants could answer “Yes” or “No,” and “What were the main reasons why you did not seek help?” which was open-ended. The responses to this open-ended question are the focus of the qualitative analysis described below. Participants completed this survey independently on paper or online, or if necessary, would have a research assistant record their exact responses.

### Analysis

Quantitative analyses were completed in SAS 9.4 (SAS Institute, Cary NC). Differences in baseline psychosocial and demographic characteristics between those who answered “Yes” and “No” to the question, “Was there ever a time when you thought you should seek help for your drinking, but you didn’t go?” were evaluated using multivariable logistic regression for categorical variables, and ANOVA for the continuous age variable. The qualitative analysis of the open-ended survey question was completed using thematic analysis methods [36, 37]. Before coding the participant responses, two authors met (CEP & NDS), reviewed the data, and created a codebook based on their assessment of the data. Codes were categorized under SEM themes that reflected why participants did not seek treatment when they felt they needed help for their alcohol use at the individual, social, or systems level. The independent coders then manually coded the responses in Microsoft Excel, and, after completion of the coding, reviewed the responses and resolved disagreements. The coders created additional codes, as needed, if the available codes did not best suit the response. If necessary, a third coder (NEC or SSC) made a final decision on any discrepancies. Exemplary quotes used in the results section below were chosen to best demonstrate the most dominant themes and codes as well as the intersectionality of codes/themes. Quotes were chosen upon consensus of all authors.

## Results

### Participant characteristics

The sample (*N* = 487) was approximately 56% male, 38% Non-Hispanic Black, 18% Hispanic, 6% Multiracial or Other Race, and had an average age of 50.0 years (Standard Deviation = 12.5). There were 129 (26%) participants who indicated a time when they thought they should seek help for their drinking but did not. Of these, 48 participants (37%) said this happened in the past year. For most sociodemographic variables, no statistically significant differences were present between those who did and did not indicate a time when they thought they should seek help for their drinking but did not (see Table 1). However, compared to those who did not feel they needed help, those who needed help but did not seek it had higher odds of at-risk drinking (OR: 2.18, 95% CI: 1.08–4.38), ever attempting to reduce their drinking (OR: 1.69, 95% CI: 1.21–7.79), and ever seeking professional alcohol treatment (OR: 11.29, 95% CI: 6.16–20.69).

**Table 1:** Participant characteristics, *n* = 487

Was there a time where you needed help for your alcohol use but did not seek it?		
	Yes (*n* = 129)	No (*n* = 358)
**Mean Age (Standard Deviation)**	50.2 (11.7)	50.0 (12.9)
**Age Categories**		
**18–34 years**^**a**^	18 (14%)	59 (16%)
**35–59 years**	78 (60%)	208 (58%)
**60 + years**	33 (26%)	91 (25%)
**Gender Identity**		
**Male**^**a**^	75 (58%)	199 (56%)
**Female**	46 (36%)	143 (40%)
**Non-Binary**	8 (6%)	15 (4%)
**Race/Ethnicity**		
**Non-Hispanic White**^**a**^	45 (35%)	140 (39%)
**Non-Hispanic Black**	54 (42%)	133 (37%)
**Hispanic**	22 (17%)	64 (18%)
**Multiracial/Other**	8 (6%)	21 (6%)
**Education**		
**Less Than High School**^**a**^	33 (26%)	75 (21%)
**High School or Equivalent**	32 (25%)	101 (28%)
**More Than High School**	64 (50%)	181 (51%)
**Employment**		
**Employed**^**a**^	36 (28%)	138 (39%)
**Not Employed**	27 (21%)	79 (22%)
**Unable to Work/Disabled**	66 (51%)	141 (39%)
**Depression Symptoms (Patient Health Questionnaire Depression Scale [PHQ-8])**		
**Depression Symptoms** **(PHQ-8 ≥ 10)**	108 (84%)	267 (75%)
**No Depression Symptoms**^**a**^ **(PHQ-8 < 10)**	21 (16%)	91 (25%)
**Anxiety Symptoms (Generalized Anxiety Disorder 7 [GAD-7])**		
**Anxiety Symptoms** **(GAD-7 ≥ 10)**	97 (75%)	209 (58%)
**No Anxiety Symptoms**^**a**^ **(GAD-7 < 10)**	32 (25%)	149 (42%)
**Drinking Status (Alcohol Use Disorders Identification Test [AUDIT-C])**		
**No Current Drinking**^**a**^ **(AUDIT-C = 0)**	28 (22%)	83 (23%)
**Current Not At-Risk Drinking** ***Assigned Male at Birth: 1 ≤ AUDIT-C < 4*** ***Assigned Female at Birth: 1 ≤ AUDIT-C < 3***	29 (22%)	153 (43%)
Current At-Risk Drinking* ***Assigned Male at Birth: AUDIT-C ≥ 4*** ***Assigned Female at Birth: AUDIT-C ≥ 3***	65 (50%)	110 (31%)
**Past-Year Drug Use** ^**b**^		
**Yes**	52 (40%)	90 (25%)
**No**^**a**^	77 (60%)	268 (75%)
**Ever Tried to Reduce Drinking?** ^**c**^		
Yes*	120 (93%)	217 (61%)
**No**^**a**^	9 (7%)	138 (39%)
**Ever Received Alcohol Treatment?** ^**c**^		
Yes*	100 (78%)	64 (18%)
**No**^**a**^	29 (22%)	294 (82%)

***Legend***: Column percentages. Percentages might not sum to 100% due to rounding and/or missing data. *p < 0.05 in multivariable logistic regression model.^*a*^*Reference.*^*b*^*Drugs used in the past year included cocaine/crack cocaine*,* heroin*,* stimulants*,* prescription opioids*,* ecstasy/MDMA*,* hallucinogens*,* poppers*,* and other drugs specified by the participant. It does not include cannabis.*^*c*^*“Ever Tried to Reduce Drinking?” means that the participant ever tried to reduce their drinking*,* either on their own or through seeking professional alcohol treatment. “Ever Received Alcohol Treatment?” means that the participant received professional alcohol treatment (i.e.*,* Alcoholics Anonymous [AA]*,* counseling or therapy*,* inpatient or outpatient treatment*,* medication*,* or self-monitoring with a device such as a breathalyzer or wrist monitor).*

### Reasons why participants did not seek help for their drinking

Of the 129 participants who did not seek help for their drinking, 112 (87%) answered the open-ended question about reasons why they did not seek help for their drinking and 13% of the sample skipped the question or provided uninterpretable responses. Forty-four of these 112 (39%) participants said this happened in the past year, while 68 (61%) said this did not happen in the past year. The most frequently reported codes were at the individual level and included Shame (*n* = 19, 17%), Denial (*n* = 14, 13%), Self-Management (*n* = 13, 12%), Not Ready (*n* = 13, 12%), Fear (*n* = 10, 9%), Did Not Want To (*n* = 10, 9%), and Did Not Think Help Would Work (*n* = 4, 4%); see Table 2 for a distribution of all 29 reported codes. Approximately 22% (*n* = 25) of participants who answered the open-ended question gave more than one reason why they did not seek alcohol treatment.

**Table 2a:** Reasons why participants did not seek help for their drinking at the individual, social, and systems levels, *n* = 112. Individual Level

Codes	General Meaning	*N* (%)
*Fear*	Afraid to seek or start treatment	10 (14%)
*Stigma*	Worried about experiencing the stigma of or judgment stemming from problem alcohol use	9 (8%)
*Guilt*	Felt guilty because of their alcohol use	2 (2%)
*Shame*	Felt ashamed or embarrassed of their alcohol use	19 (17%)
*Too Busy/Time*	Too busy or did not have the time to seek treatment	2 (2%)
*Depression*	Depression kept them from accessing treatment	2 (2%)
*Suicide*	Suicidal thoughts kept them from accessing treatment	1 (1%)
*Self-Pity*	Felt sorry for self and did not want to access treatment, self-punishment	2 (2%)
*Denial*	Denial or did not think they needed treatment	14 (13%)
*Pride*	Said that their pride led them to not seek treatment	1 (1%)
*Did Not Want To*	Did not want to receive treatment or stop their alcohol use	10 (9%)
*Liked Drinking*	Liked drinking and did not want to stop	5 (4%)
*Ambivalence*	Did not care about themselves or others or their drinking	2 (2%)
*Do It On Own*	Wanted to reduce use on own	13 (12%)
*Not Ready*	Did not feel ready or able to seek help or make a change to their drinking yet	13 (12%)
*Did Not Know How*	Did not know where to go to access treatment	3 (3%)
*Did Not Think Help Would Work*	Did not think that alcohol treatment would help so did not want to seek it	4 (4%)
*Procrastination*	Wanted to seek help later, was procrastinating seeking help	2 (2%)

**Table 2b:** Reasons why participants did not seek help for their drinking at the individual, social, and systems levels, *n* = 112. Social Level

Codes	General Meaning	*N* (%)
*Alcohol Motivation*	Used alcohol with substance use, for sex, or other purposes that led them to not want to reduce use	2 (2%)
*Social Aspect of Drinking*	Wanted to party or keep drinking with friends	2 (2%)
*Social Hindrance*	Family/friend situation kept them from seeking help	5 (4%)
*Social Support*	Did not have the social support to seek help	1 (1%)

**Table 2c:** Reasons why participants did not seek help for their drinking at the individual, social, and systems levels, *n* = 112. Systems Level

Codes	General Meaning	N (%)
*Resources*	Did not have the resources or could not access resources to stop alcohol use	6 (5%)
*Finances*	Did not have the money or was worried about loss of income	4 (4%)
*Transportation*	Did not have transportation/a way to get to alcohol treatment	3 (3%)
*Job*	Was worried about loss of job or impact to job if sought out treatment	4 (4%)
*COVID-19*	Did not seek help because of the COVID-19 pandemic	2 (2%)

***Legend***: *Column Percentages. Percentages will not sum to 100% and n’s will not sum to 112 because people could give multiple reasons why they did not seek help for their drinking.*

### Qualitative analysis

Individual reasons were reported 114 times by 91 people among the sample of 112 PWH, making it the most commonly reported theme. Accordingly, the most commonly reported reasons were at the individual level as well and included:

#### Shame

Shame (*n* = 19) was the most frequently reported reason why people did not seek care, even though they recognized they needed it. Feelings of shame also frequently co-occurred with other reasons including struggles with mental health, guilt, stigma, lack of resources, or denial.

“The shame and stigma stopped me from reaching out. Also, I knew the heavy drinking was due to my suicidal feelings and depression.” *Non-Hispanic White*,* Female*,* 51 years old*,* at-risk alcohol use.*

“I was too embarrassed to tell my doctor I have a major alcohol problem.” *Hispanic*,* female*,* 42 years old*,* at-risk alcohol use.*

“Shame and embarrassment of admitting that I have a problem and how I would be perceived.” *Non-Hispanic White*,* Female*,* 58 years old*,* at-risk alcohol use.*

#### Denial

Denial (*n* = 14) was another prominent reason why participants claimed they did not seek help for their treatment despite knowing they needed it. Some participants said that they were in denial, but in hindsight, it was clear that they were struggling with a problem. Others said they recognized that their drinking was reaching an unhealthy level but did not want to admit it to themselves or others. Many of these participants also cited other co-occurring reasons that prevented them from seeking care.

“Because I thought I didn’t need any help at the time. Feeling that I was going to be judged. Feeling that I let myself down.” *Multiracial/Other race*,* Male*,* 32 years old*,* not at-risk alcohol use*.

“Inability to face reality. Going meant acknowledging there was a problem, and I couldn’t see or admit that there was one for a long time.” *Non-Hispanic White*,* intersex/ambiguous*,* 36 years old*,* no alcohol use.*

“Because I felt like I had it under control…basically in denial at the moment.” *Non-Hispanic White*,* Female*,* 32 years old*,* at-risk alcohol use*.

#### Self-management

Some participants indicated that they thought they needed help, but wanted to do it independently rather than seek professional medical treatment (*n* = 13). Common reasons for this included thinking that their problem was not so extreme that they could not self-regulate, they had a history of reducing their use on their own, or they were continuing to use alcohol to prove to themselves that they did not have a problem.

“I wanted to do it on my own. I wanted to be sure that I was not that far gone where I would need help from a professional.” *Multiracial/Other race*,* male*,* 32 years old*,* at-risk alcohol use*.

“I knew I could cut back/stop on my own and I have been successful.” *Non-Hispanic white*,* Male*,* 61 years old*,* not at-risk alcohol use*.

“At the time I thought I had it under control and can do it on my own. I can go 8 or 9 days without drinking so I don’t consider myself an alcoholic.” *Non-Hispanic Black*,* male*,* 58 years old*,* at-risk alcohol use*.

#### Not ready

Others indicated that they knew they needed to reduce their drinking, but they were not ready (*n* = 13). Some indicated that they liked drinking and were not ready to change their behavior. Others felt that they were being forced to change, making them feel unready.

“Cause I like to drink and it’s hard to stop for me.” *Non-Hispanic Black*,* male*,* 39 years old*,* at-risk alcohol use.*

“I wasn’t ready to quit then, now I’m 3 years clean.” *Non-Hispanic Black*,* female*,* 27 years old*,* no alcohol use*.

“I felt forced, I wasn’t ready to just stop and I didn’t think I could get the right help.” *Hispanic*,* male*,* 27 years old*,* at-risk alcohol use*.

#### Fear

Fear (*n* = 10) was another common reason why participants did not seek treatment. Participants indicated fear of judgement by their healthcare providers or family/friends, fear of making a change to their behaviors, or a fear of trying to make a change to their drinking patterns and failing.

“[I] was scared to see a doctor.” *Hispanic*,* Female*,* 58 years old*,* no alcohol use*.

“[I was] afraid of failure.” *Non-Hispanic White*,* Female*,* 38 years old*,* at-risk alcohol use*.

“I was scared, I didn’t want to stop drinking.” *Non-Hispanic Black*,* Male*,* 63 years old*,* at-risk alcohol use*.

#### Did not want to

Some participants did not want to reduce their drinking (*n* = 10). Some did not want to commit to changing their behavior, while others indicated an ambivalence about seeking treatment, or that they kept changing their mind about wanting to seek alcohol treatment.

“Did not truly commit myself to wanting to stop drinking at that time.” *Multiracial/Other race*,* male*,* 44 years old*,* not at-risk alcohol use*.

“I didn’t want to go, kept changing [my] mind.” *Hispanic*,* male*,* 53 years old*,* at-risk alcohol use*.

#### Did not think help would work

Similarly, others (*n* = 4) said that they did not seek professional or medical help because they did not think it would work. This led them to try to reduce their use on their own, or not take any action either way to address their alcohol use.

“I would not trust anybody but myself. Only way to stop [is to] completely stop on [your] own will.” *Non-Hispanic White*,* male*,* 52 years old*,* no alcohol use*.

“They don’t work, if someone wants to quit, they will. Amen.” *Non-Hispanic White*,* male*,* 58 years old*,* no alcohol use*.

Social reasons for not seeking treatment despite perceived need were reported by 10 participants and systems reasons were reported by 19 participants. Four participants said that there were no reasons in particular why they did not seek treatment despite a recognized need. One participant, whose response is below, gave a list of reasons, including stigma (individual level) and lack of resources (systems level), why they did not seek help. This participant also said that they had an “unreliable history of ‘help,’” indicating that they might have had experiences with professional medical treatment that they felt did not work for them. Twenty-two percent of the participants, including some whose responses are listed above, gave more than one reason why they did not seek care. This suggests that for many there is a complicated myriad of barriers to seeking alcohol use treatment despite recognizing the need for it.

“Poverty, lack of resources, unreliable history of ‘help,’ stigma of being a drunk.” *Non-Hispanic White*,* male*,* 52 years old*,* no alcohol use.*

## Discussion

Over a quarter of this sample of PWH currently receiving HIV care in Florida indicated that, at some point in their lives, there was a time when they thought they should receive help for their drinking but they did not seek it. Though most reasons for not seeking treatment despite perceived need were at the individual level of the SEM, there was a wide variety of reasons why PWH did not seek alcohol treatment, despite a recognized need. Systems-level reasons were more commonly reported than reasons at the social level. Shame and stigma were among the most frequently reported reasons why PWH did not seek care. This finding is supported in the literature on PWH and PWOH as primary reasons why people do not seek care for many types of substance use issues [14, 16, 38–40]. Also consistent with prior literature were fear, lack of readiness to make a change, denial, wanting to do it on their own, not thinking that treatment will be effective, and a lack of finances and/or other resources [13, 15, 41–46]. A contextually novel finding of our study reported by 2 participants was the COVID-19 pandemic prevented treatment seeking, which has also been found across medical fields [47]. Surprisingly, in this sample of PWH, no participants reported any HIV-specific reasons such as drinking to cope with their diagnosis, and all the reasons reported by PWH could also apply to the general population. Past research has also found that there is no significant difference in rates of seeking AUD treatment between PWH and PWOH [12], indicating that HIV status does not necessarily worsen one’s likelihood for seeking alcohol treatment. However, it is important to investigate barriers to alcohol treatment among PWH as they could experience more negative alcohol-related harms [3–11]. Moreover, it is unknown if PWH are experiencing these barriers at a different rate than PWOH or are more impacted by specific barriers. Knowing this could allow providers and public health professionals to design treatment interventions and allocate resources appropriately to best meet the needs of PWH.

The SEM states that there are multiple levels of influence on health and behavior and that they affect and are affected by various factors and important contexts. However, there are other frameworks in which we can consider barriers to healthcare and not seeking alcohol treatment despite perceived need. In particular, the theoretical framework of the treatment seeking process [45] describes steps towards treatment seeking and allows for categorization of barriers to each step along the way. The steps in the framework include (1) problem recognition, (2) decision that change is needed, (3) decision that professional help is needed, and (4) seeking professional help, while barriers to these steps are, respectively, denial of the problem, rationalization or minimization of the problem, attempting change without professional help, and encountering insurmountable barriers to seeking professional help [45]. Participants at this study are at step 3, decision that professional help is needed, and the themes identified in this study at the individual, social, and systems levels reflect predictable steps—or commonly reported barriers—to treatment seeking.

About 22% of participants gave more than one reason for not seeking alcohol treatment. It is important to consider that this could be true for many PWH and PWOH who are struggling with unhealthy alcohol use. Therefore, only addressing one barrier at a time might not be enough to facilitate seeking treatment. When designing interventions to aid patients in receiving alcohol treatment, providers and public health professionals may consider a broad spectrum of co-occurring issues, and the most impactful interventions will likely need to address intersecting issues.

Few participants specifically cited mental health or drug co-use in their qualitative responses. Participants mentioned other reasons that often coincide with mental health such as shame, embarrassment, fear, and social issues. However, this could indicate a cognitive dissonance between participants’ experiences with mental health problems and perceived barriers to alcohol treatment. If participants are not seeking help for their alcohol use, they might also not seek help for mental health, co-occurring drug use, or other behavioral health issues.

Our study finds that shame and stigma are common indicators for not seeking treatment. While this is hard to address on a systems level, patient-care provider relationships could be strengthened to address the needs in this space. For example, providers could be trained to reduce stigma and use of stigmatizing and paternalistic language with patients. One participant in this study specifically cited not wanting to experience embarrassment by talking with their healthcare provider and addressing stigma at the provider level might help others experiencing similar barriers. Other interventions aimed at reducing stigma could also target fear or denial, such as patient education. Moreover, the AUDIT-C is underutilized as a screening tool in healthcare settings [48]. Implementing universal screening of all patients could potentially reduce stigma associated with talking about alcohol in healthcare settings. Many participants expressed a desire to reduce drinking on their own, indicating that some might not engage in a provider-driven treatment. A patient-driven, provider-supported treatment would be best in this circumstance, in which patient preferences for methods/modes of reduction are prioritized and supported, creating a locus of control for the patient.

Just as there are many definitions of alcohol recovery [49], there are many ways that people can seek treatment and engage in self-help with success. Patients might even engage in multiple methods including treatment with a trained professional, medication, and/or self-help. Providers should be aware of the range of available resources that are available to their patients, and which might best fit their needs and allow for successful drinking reduction. Greater integration of substance use treatment and HIV treatment can more effectively help those with HIV and substance use problems as PWH are subject to worse outcomes from alcohol and other substance use. Recognizing the need for help is an important primary step to substance use treatment and recovery. However, making the leap to seeking treatment could be easier with patient-centered care that acknowledges the known barriers to seeking treatment. Having greater accessibility to healthcare resources allows patients to choose the ones that best meet their needs and overcome their unique and wide-ranging barriers to care engagement.

Despite the strengths of this multi-methods study that uses qualitative and quantitative data, it is subject to several limitations. First, the qualitative data came from an open-ended survey question rather than an in-depth interview, which might have limited the responses that participants were willing to give or thought they could give. Due to the nature of the single qualitative question, further probing for a richer context could not be performed. This means that several of the reasons for not seeking care, such as stigma, could have happened at the individual, social, or systems level, but with only limited information given in the open-ended survey responses the authors used their judgment to categorize the responses. Additionally, social desirability bias could have been introduced in situations where the survey was administered by the research assistant, though the research assistants were trained to reduce this risk as much as possible. Participants were able to skip the qualitative question at will and therefore, there could be a non-response bias. Next, some qualitative responses were deemed uninterpretable due to vagueness, unclear typos, or unrelated responses. While the Florida Cohort aims to recruit a sample of PWH that is representative of PWH in the state of Florida, our results may not be generalizable to other geographic locations across the United States or in areas with greater or less care access. Given that our participants were recruited from healthcare settings and were currently receiving HIV care, our findings might also not be representative of PWH not in care. Future research should examine this question with a more extended interview to uncover the full scope of barriers to alcohol treatment and reasons why people did not seek care despite a recognized need for it. Moreover, while we know the general county where people were recruited from, we do not have information on where they lived and whether their home was specifically in a rural or urban area. Additionally, due to the nature of cross-sectional data, it is difficult to determine if participants attempted to reduce their drinking or formally sought professional alcohol treatment before or after the time in which they acknowledged needing alcohol treatment but not seeking it. We also do not have information on alcohol-related physical symptoms such as liver fibrosis staging, obesity, or other alcohol-related health outcomes. The SEM has a long history of use in qualitative research, but a limitation is that individuals tend to add more weight to personal beliefs and motivations as impacting their health and behaviors and less weight to systems-level factors, which could play a larger role in inhibiting healthcare engagement. The use of a different framework might have reduced potential bias in the results. Finally, a “No” response to the survey question used in this study only captured the perception of needing help with alcohol and not seeking it: therefore, a “No” response could mean the participant never thought they had a problem with alcohol use, or they did think they had a problem with alcohol use and they sought help. For the purposes of this study, we are only interested in those who perceived they had a problem with alcohol use and recognized they needed help but did not seek it. We sought to understand their motivations for not seeking care despite their perceived need, so the “No” responses were outside of the research scope of interest. Further, there may have been participants that truly needed help with their drinking but did not perceive that need. It is possible that those that objectively needed help reducing alcohol, but did not perceive the need could have had different types of barriers to receiving alcohol treatment.

## Conclusions

Many participants endorsed not seeking alcohol treatment despite a perceived need among this sample of PWH in Florida with lifetime drinking. Participants who did not seek treatment despite perceived need were more likely to endorse at-risk drinking and a history of trying to reduce drinking and seeking alcohol treatment. The most common reasons participants did not seek care were all at the individual level: shame, denial, wanting to do it on their own, not feeling ready, fear, and not wanting to seek care. However, PWH experienced barriers at the individual, social, and systems levels that kept them from seeking alcohol treatment. To address these barriers, alcohol treatment strategies should be accessible, based in harm reduction, and tailored to meet the needs of PWH in order to help them achieve their goals.

## Data Availability

The data used during the current study can be requested from the Southern HIV and Alcohol Research Consortium (https://sharc-research.org/research/data/sharc-concepts-system/).

## Abbreviations

ART: Antiretroviral therapy
AUD: Alcohol Use Disorder
AUDIT-C: Alcohol Use Disorders Identification Test
GAD-7: Generalized Anxiety Disorder-7
PHQ-8: Patient Health Questionnaire
PWH: People with HIV
PWOH: People without HIV
SEM: Social-Ecological Model
